# Point-of-care detection of SARS-CoV-2 antigen among symptomatic vs. asymptomatic persons: Testing for COVID-19 vs. infectivity

**DOI:** 10.3389/fpubh.2022.995249

**Published:** 2022-10-17

**Authors:** Karin Neukam, Alicia Lucero, Alicia Gutiérrez-Valencia, Lucas Amaya, Natalia Echegoyen, Antonella Martelli, Cristina Videla, Federico A. Di Lello, Alfredo P. Martínez

**Affiliations:** ^1^Unit of Infectious Diseases, Microbiology and Preventive Medicine, Virgen del Rocío University Hospital, Seville, Spain; ^2^Instituto de Biomedicina de Sevilla, University of Seville, Consejo Superior de Investigaciones Científicas (CSIC), Seville, Spain; ^3^Virology Section, Centro de Educación Médica e Investigaciones Clínicas Norberto Quirno “CEMIC”, Buenos Aires, Argentina; ^4^Facultad de Farmacia y Bioquímica, Instituto de Investigaciones en Bacteriología y Virología Molecular (IBaViM), Universidad de Buenos Aires, Buenos Aires, Argentina; ^5^Consejo Nacional de Investigaciones Científicas y Técnicas (CONICET), Buenos Aires, Argentina

**Keywords:** SARS-CoV-2, COVID-19, point-of-care, rapid antigen testing, PCR, public health, viral kinetics, surveillance

## Abstract

**Background:**

Management of the coronavirus disease 2019 (COVID-19) pandemic caused by a novel severe acute respiratory syndrome coronavirus-2 (SARS-CoV-2) requires rapid and simple methods to detect COVID-19 patients and identify potential infectors. This study aimed to evaluate the utility of a point-of-care (PoC) rapid antigen diagnostic test (Ag-RDT) in these settings.

**Patients and methods:**

Individuals who consecutively presented for SARS-CoV-2 testing at a tertiary care center in Buenos Aires, Argentina, underwent PoC Ag-RDT testing and real-time RT-PCR (qRT-PCR) on the same day during June 2021.

**Results:**

Of 584 included subjects, 108 (18.5%) were symptomatic for COVID-19 while the remaining presented for miscellaneous reasons unrelated to possible or confirmed contact with a SARS-CoV-2-infected individual. A positive Ag-RDT result was obtained in 26 (24.1%) symptomatic and 7 (1.5%) asymptomatic persons (*p* < 0.001), which was concordant with qRT-PCR in 105/108 [97.2%, Cohen's kappa coefficient (κ) = 0.927] symptomatic and 467/476 (98.1% κ = 0.563) asymptomatic participants, with a positive percentage agreement (PPA; 95% confidence interval) of 89.7% (71.5–97.3%) and 42.9% (18.8–70.4%), respectively. None of the 11 false-negative diagnoses showed a C_t_-value ≤20. Considering only failures with a C_t_-value below 31 as hypothetical infectivity threshold of 10^5^ SARS-CoV-2 RNA copies/mL, concordance was observed in 98.1% (κ = 0.746) in the asymptomatic population, accounting for a PPA of 66.7% (30.9–91%).

**Conclusions:**

PoC Ag-RDT accurately detected active SARS-CoV-2 infection and showed acceptable diagnostic performance in asymptomatic persons potentially spreading infectious virus. Ag-RDT may therefore be useful to slow down or stop transmission by enabling adequate decisions on isolation at a public health level.

## Introduction

The ongoing (coronavirus disease 2019) COVID-19 pandemic caused by novel severe acute respiratory syndrome coronavirus-2 (SARS-CoV-2) has taught the importance of both early diagnosis of symptomatic patients (1) to ensure medical care for COVID-19 patients, as well as the implementation of rapid public health measures in order to limit or halt transmission (2). It is estimated that up to 45% of the SARS-CoV-2 infected people do not develop symptoms (3–6), yet asymptomatic transmission may occur (7–10). Therefore, surveillance of allegedly healthy persons and screening of at-risk populations represent essential constituents of curbing the pandemic. This sudden and urgent request of SARS-CoV-2 diagnostic devices for different settings was settled through multiple emergency use authorizations (11).

Detection of viral RNA in nasopharyngeal swabs (NPS) by nucleic acid amplification test (NAAT), such as quantitative reverse transcription (qRT-) polymerase chain reaction (PCR), represents the current standard for the diagnosis of SARS-CoV-2 infection. However, PCR techniques are costly, time-consuming and require both advanced operator training and laboratory infrastructure (12, 13). Rapid antigen diagnostic tests (Ag-RDT) represent an alternative suitable to be conducted at points-of-care (PoC) as they are considerably economic, easy-to-use, and provide rapid results (14). Most Ag-RDTs shows an overall specificity close to 100% while the sensitivity varies widely from ~70–100%. Sensitivity increases substantially when tests are applied in the first 5–7 days upon symptoms onset (13–16), which coincides with SARS-CoV-2 peak viral load in the upper respiratory tract (17). However, the accuracy of firstly available Ag-RDT remains below the reference standard NAAT (ECSMIDS).

The BD Veritor Ag-RDT is based on a chromatographic digital immunoassay that qualitatively detects SARS-CoV-2 nucleocapsid antigen from nasal swabs within 15 min (18). It shows adequate diagnostic performance (19), a high user friendliness (20) and in July 2020, an emergency use authorization was issued for its use to diagnose SARS-CoV-2 infection in people showing symptoms compatible with COVID-19 ≤5 days upon onset (21). However, given the low experience and information gaps, controversy arouse on the adequacy of first- and second-generation Ag-RDT as tool to diagnose COVID-19 in people without respiratory infection symptoms (22, 23) and many Ag-RDT, including Veritor, remain to be clinically validated in the asymptomatic population (18, 19). Still, testing of asymptomatic individuals is recommended in some settings (24) and NAAT techniques may not represent an appropriate standard reference in this population as they do not distinguish infectious and non-infectious virus, thus failing to identify persons with non-infectious SARS-CoV-2 infection in whom isolation could be spared. In contrast, recent studies suggest an association between positive Ag-RDT results and cell culture positivity, as well as the presence of subgenomic RNA, a surrogate for the presence of viable virus (25, 26).

The aim of this study was to determine the diagnostic performance and to evaluate the clinical implication of the Veritor Ag-RDT when applied in symptomatic vs. asymptomatic individuals in a real-life setting.

## Materials and methods

### Study design and population

A cross-sectional study was conducted at the Center for Medical Education and Clinical Research “Norberto Quirno” (CEMIC), Buenos Aires, Argentina. From June 1st to 30th 2021, all subjects ≥18 years old who consecutively underwent testing for SARS-CoV-2 infection were recruited. Subjects may have been attended due to (i) presence of one or more symptoms compatible with COVID-19 during five days or less or (ii) other, non-SARS-CoV-2-related, circumstances such as pre-surgical evaluation, intended traveling, returning to restricted activities, or learning about their infection status while being asymptomatic for COVID-19. After explaining the protocol, the candidates were invited to join the study and upon acceptance, they were asked to give their written informed consent.

### Laboratory determinations

SARS-CoV-2 Ag-RDT (index test): Antigen testing was conducted from anterior nasal swabs by the Veritor™ System for Rapid Detection of SARS-CoV-2 (Becton, Dickinson and Company, Franklin Lake, NJ, USA). This test has a limit of detection of 140 50% tissue culture infective doses of nucleocapsid protein and was used according to the manufacturer's instructions (18). Briefly, swabs were added to extraction reagent tubes at room temperature within 60 min after sampling and mixed for at least 15s. Three drops extraction buffer/specimen mixture were then added to a test cartridge. After 15 min of incubation, the test devices were interpreted using a Veritor™ Analyzer.

RT-PCR (reference standard): RNA was extracted from NPS and purified using GenePure Pro, Nucleic Acid Purification System (Bioer Technology, Hangzhou, China). qRT-PCR was performed using the RealStar^®^ SARS-CoV-2 RT-PCR kit 1.0 (Altona Diagnostics, Hamburg, Germany) on a Roche Cobas z 480 RT-PCR device.

### Statistical analysis

Descriptive statistics were performed, expressing categorical values as number (percentage) and continuous variables as median (IQR). The outcome variable was the proportion of correct (true-positive and true-negative) Ag-RDT results as confirmed by qRT-PCR. For comparison of categorical variables, the Chi-square or the Fisher's tests were applied while continuous variables were analyzed by means of the Student's *t*-test or the Mann-Whitney U test. In order to assess the agreement between Ag-RDT and qRT-PCR outcomes, Cohen's kappa coefficient (κ) was calculated. Diagnostic performance was evaluated by calculating the positive (PPA), negative (NPA) and overall (OPA) percent agreement, as well as, the positive (PPV) and negative (NPV) predictive value with their respective 95% confidence interval (95CI). Statistical analyses were carried out using the SPSS statistical software package release 23.0 (IBM SPSS Inc., Chicago, IL, USA) and STATA 9.0 (StataCorp LP, College Station, TX, USA).

### Ethical aspects

The study was designed and performed according to the Helsinki declaration and all blood donors gave their written informed consent (Study protocol EX-2021-06438339-UBA DME#SSA_FFYB, Ethics committee of the Facultad de Farmacia y Bioquímica, Universidad de Buenos Aires).

## Results

### Study population

**Figure 1:**
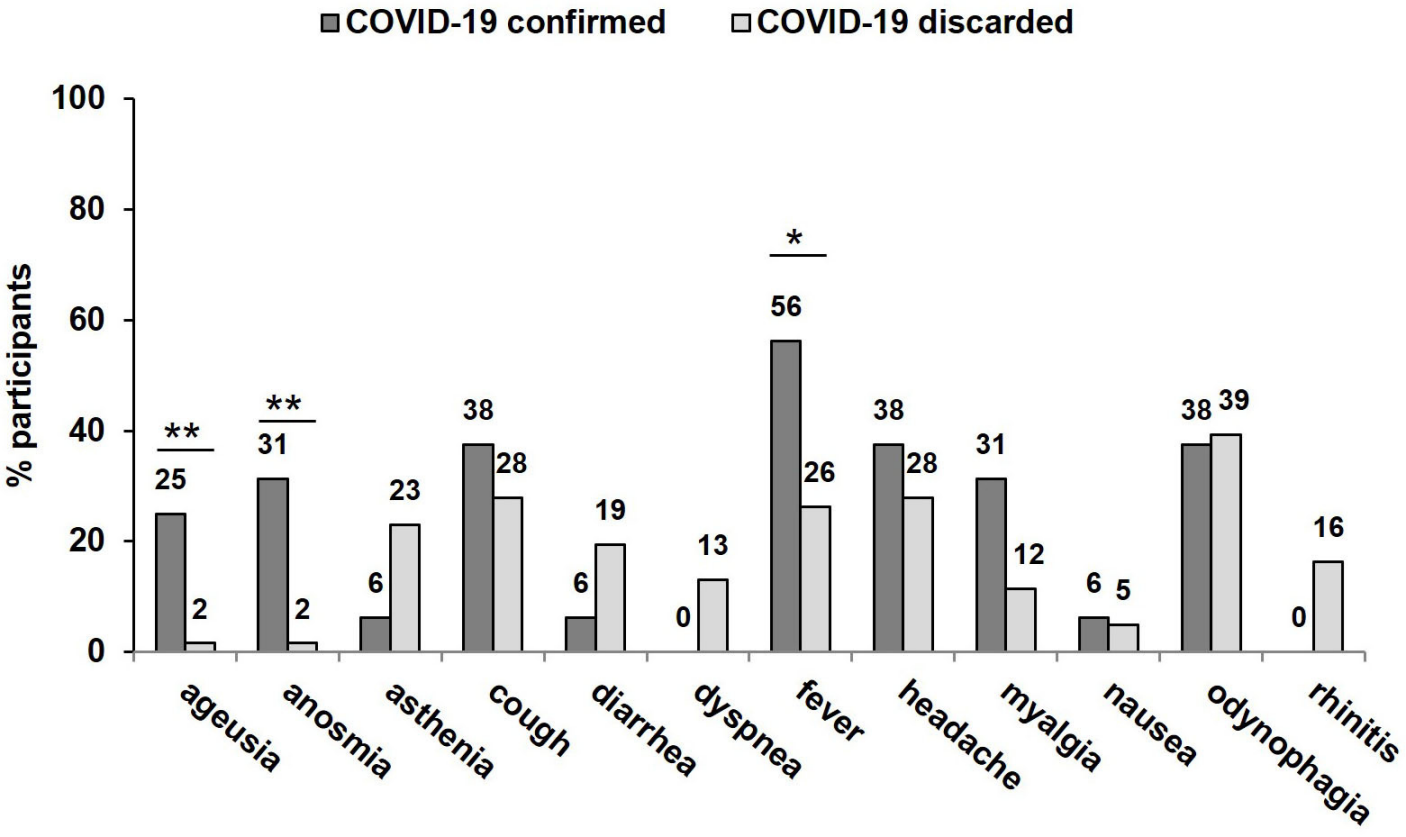
Proportions of different symptoms as reported at the day of testing for SARS-CoV-2-infection stratified for the presence of COVID-19. *p < 0.05; **p < 0.01.

A sample of 584 subjects was included in the study, 108 (18.5%) participants were symptomatic for COVID-19 while the remaining 476 (81.5%) did not present COVID-19-related symptoms. Thirty-one (41.9%) presented with only one symptom, 43 (58.1%) with ≥2, and 29 (39.2%) with ≥3. Among the 358 (61.3%) female participants, 61 (56.5%) were symptomatic and 297 (62.4%) were asymptomatic, *p* = 0.152. The proportions of the different symptoms are shown in Figure 1. The median (IQR) age was 43 (33–55) years, corresponding figures for those with and without symptoms were 46 (33–67) and 42 (33–54) years, *p* = 0.045, respectively.

### Testing agreement and diagnostic performance

A positive qRT-PCR result was obtained in 29 (26.9%) individuals who presented with symptoms and in 14 (2.9%) who did not (*p* < 0.001), accounting for 7.4% overall prevalence of SARS-CoV-2 infection in the study population. In symptomatic individuals, Ag-RDT testing yielded 26 (24.1%) positive results, while among the asymptomatic, 7 (1.5%) subjects tested positive (*p* < 0.001). Results from Ag-RDT and reference qRT-PCR were concordant in 572/584 (97.9%; κ = 0.831) determinations: 105/108 (97.2%; κ = 0.927) among symptomatic individuals and 467/476 (98.1% κ = 0.563) among the asymptomatic participants. Only one (8.3%) out of the 12 discordant diagnoses was false-positive. None (0%) of the individuals who presented with more than one symptom had a discordant result. Detailed diagnostic performance is shown in Table 1.

**Table 1:** Diagnostic performance of the BD Veritor System for Rapid Detection of SARS-CoV-2.

**Population**	**Prevalence**	**PPA**	**NPA**	**OPA**	**PPV**	**NPV**
	% (n/N)^*^	**(95% CI)**	**(95% CI)**	**(95% CI)**	**(95% CI)**	**(95% CI)**
Overall	5.7	74.4	99.8	97.9	97	98
	(33/584)	(59.8–85.1)	(99–100)	(96.4–98.8)	(82.5–99.8)	(82.5–99.8)
Symptomatic	24.1	89.7	100	97.2	100	96.3
	(26/108)	(73.6–96.4)	(95.4–100)	(92.2–99.1)	(84–99.7)	(88.9–99.1)
Asymptomatic	1.5	42.9	99.8	98.1	85.7	98.3
	(7/476)	(21.4–67.4)	(98.8–100)	(96.4–99)	(42–99.3)	(96.5–99.2)
C_t_ < 31, overall	6.2	88.9	99.8	99.1	97	99.3
	(36/577)	(74.7–95.6)	(99–100)	(98–96.6)	(82.5–99.8)	(98–99.8)
C_t_ < 31,	25.5	96.3	100	99.1	100	98.8
symptomatic	(27/106)	(81.7–99.3)	(95.4–100)	(94.8–99.8)	(84–99.7)	(92.3–99.9)
C_t_ < 31,	1.9	66.7	99.8	99.2	85.7	99.4
asymptomatic	(9/471)	(30.9–91)	(98.6–100)	(97.8–99.7)	(42–99.3)	(98–99.8)

PPA, positive percent agreement; NPA, negative percent agreement; OPA, overall percent agreement; PPV, positive predictive value; NPV, negative predictive value; CI, confidence interval; C_t_, cycle threshold.

Standard reference: RealStar SARS-CoV-2 real-time RT-PCR.

* Based on real-time RT-PCR.

### Factors potentially influencing diagnostic performance

**Figure 2:**
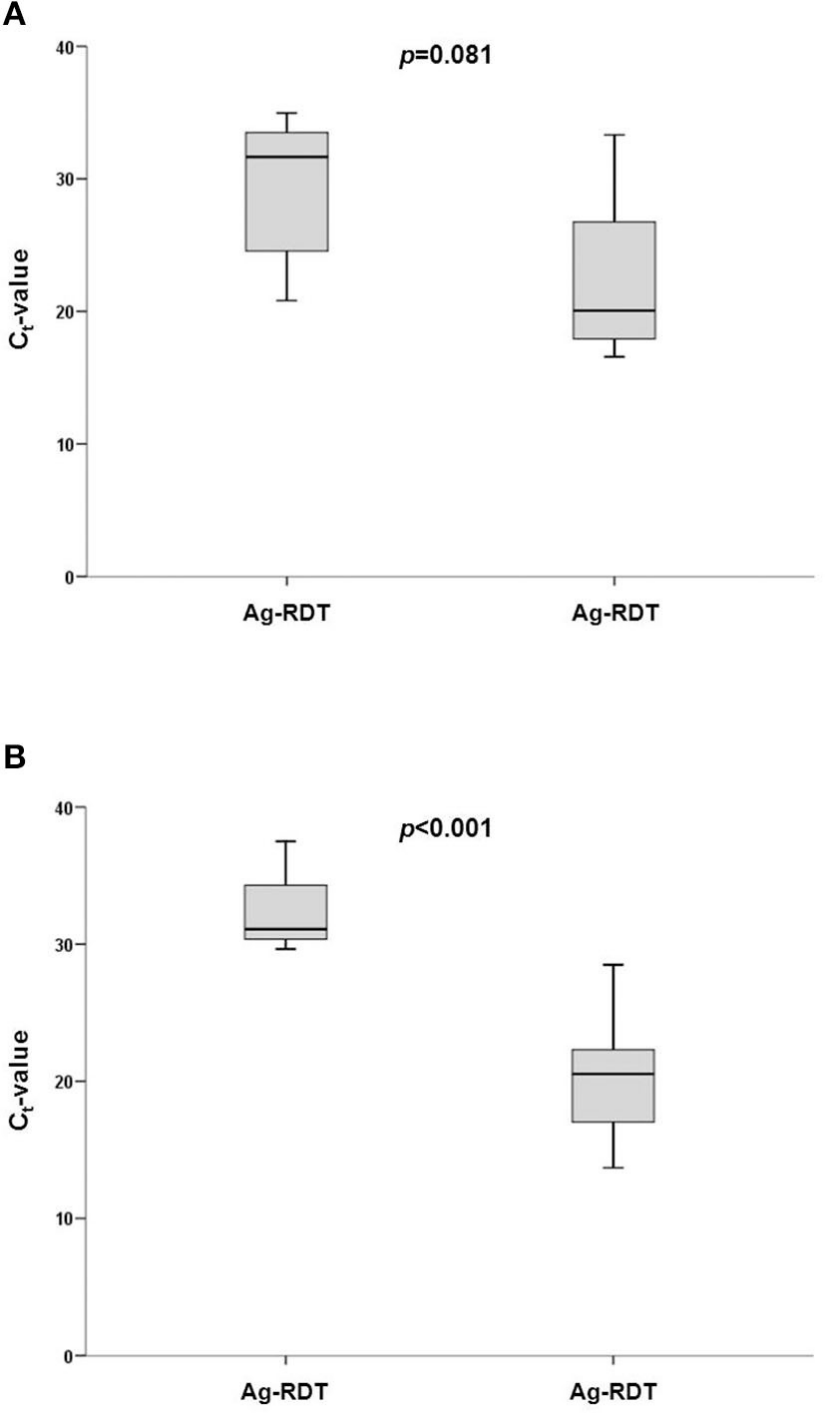
Cycle threshold (C_t_)-values observed for SARS-CoV-2 determination by qRT-PCR according to agreement with BD Veritor System for Rapid Detection of SARS-CoV-2 (Veritor) in **(A)** asymptomatic and **(B)** symptomatic participants.

Ag-RDT results were not in agreement with qRT-PCR in 8 (2.2%) women and 4 (1.8%) males, *p* = 0.774. Median age among cases with a discordant vs. a concordant result was 43.4 (33.1–63.3) and 23 (33–54.9) years, respectively, p = 0.560. Overall median (IQR) qRT-PCR cycle threshold (C_t_-values) were 21.8 (17.9–28.5): 21.6 (17.1–24.3) vs. 26.6 (20.2–33.2) for samples derived from symptomatic and asymptomatic participants (*p* = 0.034). Figure 2 shows the corresponding C_t_-values stratified for testing agreement and the presence of symptoms. None of the 11 false-negative diagnoses by Ag-RDT showed a C_t_-value ≤20 while 5 (45%) had a C_t_-value ≥33. Considering only failures with a C_t_-value below 31, accounting for a SARS-CoV-2 viral load of 10^5^ copies/mL (27), overall agreement between Ag-RDT and qRT-PCR was observed in over 99% (κ = 0.923), being almost perfect among those with symptoms (κ = 0.975) and substantial in those without (κ = 0.746), respectively. The diagnostic performance in the overall population and the subgroups is presented in Table 1.

## Discussion

### Summary

The present study confirms the accuracy of a commercially available PoC Ag-RDT to identify COVID-19 in a real-life setting of patients showing a flu-like symptomatic profile, which is crucial when rapid diagnosis is required for taking emergency clinical management decisions. Furthermore, in those without known prior contact with a SARS-CoV-2 index case and who were asymptomatic for COVID-19, the Ag-RDT showed a good clinical performance for C_t_-values below a theoretical infectivity threshold.

### Main discussion

#### Ag-RDT testing in asymptomatic subjects

While current guidelines strongly recommend the use of NAAT vs. Ag-RDT testing in individuals with symptoms compatible with severe or critical illness, or symptomatic persons at high risk for a severe course of infection, there is only a weak recommendation against Ag-RGT use in those with moderate symptoms, disregarding the age and the date of system onset (22). As these recommendations are based on a very low certainty of evidence, Ag-RGT testing is considered an alternative when NAAT are not available (23). In asymptomatic individuals at risk of exposure, testing is recommended in various settings (24) however, recommendations on the testing strategy is inconsistent or no recommendation is given due to the lack of evidence (22, 23). Thus, the benefits of PoC Ag-RDT at a population health level remain uncertain as available data is scarce and inconsistent, mainly due to deficient study designs (28). Even less is known about their performance in subjects who did not have contact with an index case and who are therefore likely to show a lower prevalence of SARS-CoV-2 infection as compared to symptomatic individuals, which positively correlates with test sensitivity (29). The considerably low sensitivity of Ag-RDTs observed in asymptomatic individuals (13) represents a major drawback. Indeed, in the present study, an overall PPA of 42.9% in the asymptomatic population, which lies within the considerably wide sensibility range previously reported for Veritor (30–33). However, it is worth to note that these low values are derived from comparisons with RT-PCR, in which a positive result does not necessarily imply infectiveness, since NAAT does not distinguish inactive from viable virus. Although shedding of SARS-CoV-2 RNA and consequently positive RT-PCR results may persist over weeks (17), data on cell culture of respiratory tract isolates suggest that infectious virus are only present during the first 8–12 days (34–37). It is estimated that, in general, viral shedding exceeds shedding of viable virus up to 45 days, whereas infectious virus may be present up to 6 days prior to symptom onset. Hence, an unsurprisingly low agreement between RT-PCR and cell culture has been found (34). In contrast, sensitivity roughly doubled from 24–50 to 50–82% when matched with cell culture (38) and the herein applied Veritor test in particular showed a PPV of 90% using viral culture as standard reference (34). This finding was confirmed in a recent study, in which Veritor showed a sensitivity of 74% when compared to cell culture (25). Similarly, a study on the diagnostic performance of different Ag-RGT by contrasting the results with detection of subgenomic RNA suggests their ability to determine the presence or absence of replication-competent, thus potentially transmissible, virus (26). In order to implement these findings to the clinical practice, one should take into account the role of infectivity. While various studies have investigated the infectivity of SARS-CoV-2 viral load, no standard threshold has been established. A relation between the C_t_-value and cell culture positivity has widely been reported (7, 36, 37, 39, 40), with an observed decrease by 0.32–0.67 in the estimated odds ratio of recovering infectious virus with each unit increase in C_t_-value (37, 40). In accordance with these findings, in the present study, the sensitivity of the Ag-RDT among asymptomatic participants increased more than 1.5-fold when only failures with C_t_-values below 31 were considered, which is also below the Veritor Ag-RDT median C_t_-value of 32 for false-negative results in asymptomatic individuals compared to NAAT, according to a recent study (32). Likewise, this threshold is within the range of previous reports where a low or no proportion of positive viral cultures were obtained for C_t_-values varying from 28 to 34 (7, 36, 39). This variability may be explained by the fact that the C_t_-value is subject to the RT-PCR parameters and comparisons between studies using it as surrogate for viral load have to be drawn with caution (41). Taken into account the RT-PCR assay applied in the present work, a C_t_-value of 31 corresponds to a viral load of approximately 10^5^ copies/mL (27). To note, this value is one to two orders of magnitude below the hypothetical infectiousness threshold range of 10^6^-10^7^ copies/mL as the *in-vitro* isolation of replicative virus suggests (40, 42, 43). Also, it corresponds to the range of viral load below which the sensitivity of the Veritor test becomes almost zero when compared to viral culture, as reported recently (25). In accordance, in a large contact tracing study, ~15 vs. 50% RT-PCR-positive contacts were identified when index cases presented a viral load below 10^4^ vs. 10^6^ copies/ml, respectively (44). Taken together, the Ag-RDT showed moderate performance in detecting SARS-CoV-2 infection amongCOVID-19 asymptomatic persons with a low prevalence of infection in a real-life setting. However, rather than detecting infection, the test may be useful to rapidly identify potential infectors during their viral load peak, who should be isolated and, at the same time, it may be able to spare confinement for those who are not infectious. Based on these findings, large prospective studies should be designed in order to confirm this issue.

#### Ag-RDT testing in symptomatic subjects

In symptomatic individuals, an adequate diagnostic performance of many but not all commercially available Ag-RDT to detect SARS-CoV-2 infection has been demonstrated in various studies (13, 15, 16). Still, reports on the sensitivity Ag-RDT vary wildly. In the present study with a considerably high sample size, a sensitivity of 89.7 % (CI: 71.5–97.3%), with only 3 out of 26 false-negative results, and a specificity of 100 % (CI: 94.2–99.9%) was observed in symptomatic patients during the first 5 days of symptom onset. A similar performance has been reported in real-life studies, where sensitivities from 76% to over 90% were observed (20, 30, 33, 45). Likewise, a study on Veritor meeting the FDA criteria for emergency use authorization approval found a sensitivity of 82–88% during the first 6 days of symptom onset. The herein presented results therefore confirm the accuracy of the Veritor Ag-RDT for diagnosing COVID-19 in the early stage of symptomatic infection.

#### Symptoms of COVID-19

Another obstacle to ensure effective surveillance represents the identification of symptoms compatible with COVID-19. While the symptoms reported by the participants in this study were consistent with COVID-19 (1), the majority were also compatible with infection by influenza viruses (1, 18, 46). Of the individual symptoms, only ageusia, anosmia and, to a lesser extent, fever were significantly associated with confirmed COVID-19, which is in accordance with previous reports (46), while symptoms such as asthenia, diarrhea, and rhinitis were not. The similarity of COVID-19-related complex with influenza virus infection may cause actual SARS-CoV-2 infected persons to weigh up whether to present for testing. The possibility of obtaining a result fast and in a setting that is easy to access, together with the widely unconsidered fact that anterior nasal sampling results in far less objection than NPS required for NAAT, adds up to a higher probability of individuals actually undergoing a test when showing symptoms, therefore lowering the rate of undiagnosed infection. To note, this is also probably even more the case for asymptomatic testing.

#### Age and gender

In the present study, age and gender did not impact in the agreement between the Ag-RDT and RT-PCR results, which is in accordance with previously reported data (27). This is not surprising since no clinically relevant differences in SARS-CoV-2 viral loads according to age (47, 48) and gender (48) have been described. A mild age impact has been reported in a study on the use of Veritor Ag-RDT in self-sampling testing, however, the authors hypothesize that this is likely attributable to lower sampling skills rather than the age itself (49).

### Limitations

The main limitation of this study is that cell culture and RT-PCR calibration were not done in-house. To date, a considerable gap in understanding the viral dynamics, infectiousness, and prevention of disease spread remains, especially in this rapidly changing setting including emergent viral variants with a different infection profile and the availability of vaccines. Randomized clinical trials and large, prospective real-life studies are warranted to confirm the role of Ag-RDT as discussed herein. Additionally, no second test in asymptomatic persons was conducted as proposed by the manufacturer due to the lack of validation studies in asymptomatic individuals (18). The analysis of consecutive results may have been interesting as another study on the Veritor test described a positive rate of 63% 2 weeks after a false-negative determination (27). However, this study did not include follow-up visits in order to keep the burden for the patients as low as possible.

### Final conclusion

In conclusion, the PoC Ag-RDT showed a good performance in diagnosing COVID-19 in patients during the first days of symptom onset, allowing rapid medical care and isolation measures when molecular techniques are not available. However, prevention measures of viral spread to slow down or stop the pandemic cannot be limited to the symptomatic population. The use of economic, rapid, and simple Ag-RDT may be an appropriate approach for large-scale surveillance and screening conducted in remote settings in order to identify individuals who are likely to be shedding infectious virus, which would otherwise go unnoticed. Facilitating decisions on measures regarding isolation and contact tracing could represent an essential tool for public health management of the COVID-19 pandemic.

## Data availability statement

The raw data supporting the conclusions of this article will be made available by the authors, without undue reservation.

## Ethics statement

The studies involving human participants were reviewed and approved by Ethics Committee of the Facultad de Farmacia y Bioquímica, Universidad de Buenos Aires. The patients/participants provided their written informed consent to participate in this study.

## Author contributions

KN, FD, and APM: study design, conception, and drafting of the manuscript. LA, NE, AM, and CV: data collection. KN, AL, FD, and APM: data analysis and interpretation. All authors provided intellectual content of critical importance to the work described, read, and agreed to the published version of the manuscript.

## Funding

This work was supported by the Instituto de Salud Carlos III, co-financed by the European Development Regional Fund (A way to achieve Europe), Subprograma Miguel Servet (grant numbers CP19/00159 to AG-V and CPII18/00033 to KN). FD is a member of the National Research Council (CONICET) Research Career Program. KN is the recipient of a Senior Researcher Contract by the Office for Families and Health of the Andalusian Council (RH-0019-2021).

## Conflict of interest

The authors declare that the research was conducted in the absence of any commercial or financial relationships that could be construed as a potential conflict of interest.

## Publisher's note

All claims expressed in this article are solely those of the authors and do not necessarily represent those of their affiliated organizations, or those of the publisher, the editors and the reviewers. Any product that may be evaluated in this article, or claim that may be made by its manufacturer, is not guaranteed or endorsed by the publisher.
